# MRI Feature-Based Nomogram Model for Discrimination Between Non-Hypervascular Pancreatic Neuroendocrine Tumors and Pancreatic Ductal Adenocarcinomas

**DOI:** 10.3389/fonc.2022.856306

**Published:** 2022-05-19

**Authors:** Jiake Xu, Jie Yang, Ye Feng, Jie Zhang, Yuqiao Zhang, Sha Chang, Jingqiang Jin, Xia Du

**Affiliations:** ^1^ Department of Gastroenterology, Kunshan Second People’s Hospital, Kunshan, China; ^2^ Department of Radiology, The Affiliated Hospital of Guizhou Medical University, Guiyang, China; ^3^ Department of Radiology, Kunshan Second People’s Hospital, Kunshan, China

**Keywords:** pancreatic neuroendocrine tumors, pancreatic ductal adenocarcinoma, magnetic resonance imaging, nomogram, non-hypervascular

## Abstract

This study aimed to investigate whether magnetic resonance imaging (MRI) features could differentiate non-hypervascular pancreatic neuroendocrine tumors (PNETs) from pancreatic ductal adenocarcinomas (PDACs). In this study, 131 patients with surgically and pathologically proven non-hypervascular PNETs (*n* = 44) or PDACs (*n* = 87) were enrolled. Two radiologists independently analyzed MRI imaging findings and clinical features. Relevant features in differentiating non-hypervascular PNETs from PDACs were identified *via* univariate and multivariate logistic regression models. The MRI feature-based nomogram was constructed based on multivariable logistic analysis and the reliability of the constructed nomogram was further validated. The results showed that tumor margin (*P* = 0.012; OR: 6.622; 95% CI: 1.510, 29.028), MPD dilation (*P* = 0.047; OR: 4.309; 95% CI: 1.019, 18.227), and signal in the portal phase (*P* < 0.001; OR: 53.486; 95% CI: 10.690, 267.618) were independent discriminative MRI features between non-hypervascular PNETs and PDACs. The discriminative performance of the developed nomogram was optimized compared with single imaging features. The calibration curve, C-index, and DCA validated the superior practicality and usefulness of the MRI-based nomogram. In conclusion, the radiologically discriminative model integrating various MRI features could be preoperatively and easily utilized to differentiate non-hypervascular PNETs from PDACs.

## Introduction

Pancreatic neuroendocrine tumors (PNETs) are rare and heterogeneous pancreatic tumors, which arise from pancreatic neuroendocrine cells (1, 2). PNETs have various clinical behaviors and account for about 2%–10% of all pancreatic neoplasms (3). With the development of imaging technology, the detection and diagnosis rates of PNETs have been increasing in recent years (4).

The common imaging features of PNETs have been summed up as a hypervascular and well-defined solid pancreatic mass, which are exhibited as relatively hyperenhancement in the arterial phase of computed tomography (CT) or magnetic resonance imaging (MRI) (4, 5). Nevertheless, several studies showed that up to 42% of PNETs exhibit non-enhancement in the arterial phase (6). Therefore, such overlapping imaging features between hypovascular PNETs and low-enhancement pancreatic ductal adenocarcinomas (PDACs) make it difficult to preoperatively distinguish the two tumors on imaging. Remarkably, PDACs have a relatively worse prognosis and lower survival rates and, more importantly, lower resectability rate with a wider range of excision compared with PNETs (7). Therefore, it is of great clinical significance to preoperatively discriminate between PNETs and PDACs.

Recently, several studies have focused on the difference of CT imaging features between non-hypervascular PNETs and PDACs (8, 9). For instance, Karmazanovsky et al. have reported that a series of CT imaging features, such as a well-defined margin, morphologic characteristic, and enhancement pattern, contribute to differentiate non-hypervascular PNETs from PDACs (8). Moreover, Xue’s group has constructed a combined model, integrating CT-based radiomics signature and clinical–radiological features and exhibiting a better performance on the discrimination between atypical non-functional neuroendocrine tumors and PDACs (10). Unfortunately, few studies have reported the value of MRI features in discriminating non-hypervascular PNETs from PDACs. Due to its superior assessment performance for pancreatic parenchyma, pancreatic ducts, and peripancreatic soft tissues or vessels (11), MRI may be helpful in improving the differential diagnosis of non-hypervascular PNETs from PDACs. Moreover, to our knowledge, the radiological identification model integrating various MRI features for differentiating non-hypervascular PNETs from PDACs has not been reported.

Therefore, the purpose of our study was to evaluate whether MRI features are helpful to differentiate non-hypervascular PNETs from PDACs. Furthermore, we tried to develop a radiological identification model integrating significant MRI features for the precise differentiation of non-hypervascular PNETs from PDACs.

## Materials and Methods

### Patient Selection

**Figure 1:**
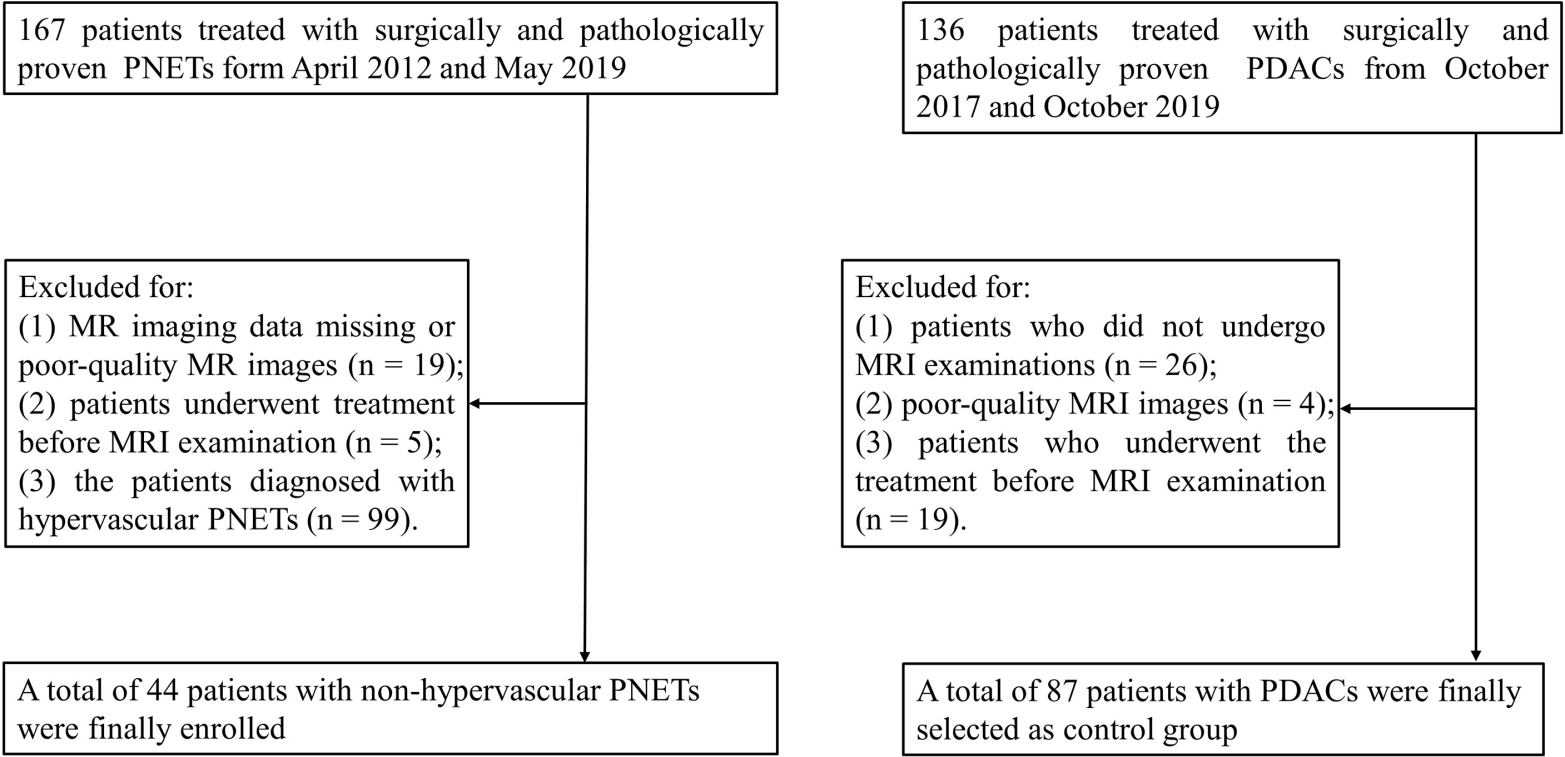
Flowchart of the selection of patients with non-hypervascular pancreatic neuroendocrine tumors (PNETs) and pancreatic ductal adenocarcinomas (PDACs).

This retrospective study was approved by the Institutional Review Board of the Affiliated Hospital of Guizhou Medical University and the requirement for informed consent was waived. Between April 2012 and May 2019, all patients with surgically and pathologically proven PNETs in our hospital were enrolled. The inclusion criteria were as follows: a) patients who underwent MRI examination with a standardized MRI protocol within 2 weeks before surgery and b) patients who did not receive local or systemic treatment prior to surgery. The exclusion criteria, on the other hand, were as follows: a) missing MR imaging data or poor-quality MR images, b) patients underwent treatment before MRI examination, and c) patients diagnosed with hypervascular PNETs. The detailed data are presented in **Figure 1**. Hypervascular PNETs were defined as tumors that exhibited hyperintensity in the MRI arterial phase compared with the adjacent normal pancreas parenchyma. On the other hand, non-hypervascular PNETs are considered atypical tumors. All PNETs in this study were defined by three experienced reviewers. Finally, 44 patients with non-hypervascular PNETs were enrolled in this cohort.

In addition, 136 consecutive patients with surgically and pathologically proven PDACs between May 2018 and October 2019 were enrolled as a comparison group. Among these cases, 49 patients were excluded for the following reasons: a) patients did not undergo MRI examinations (*n* = 26), b) patients’ MRI images were of poor quality (*n* = 4), and c) patients underwent treatment before the MRI examination (*n* = 19).

### MRI Protocol

All cases in this study were examined by contrast-enhanced MRI in a 1.5-T MRI scanner (Magnetom Aera; Siemens Medical Solutions, Germany) with a standardized scan protocol. The examination protocol is as follows: fat-suppressed T2-weighted two-dimensional turbo spin-echo (TSE) and diffusion-weighted imaging (DWI) with *b*-values = 0 and 500 s/mm^2^ utilizing respiratory triggering. Three-dimensional T1-weighted volumetric interpolated breath-hold examination (VIBE) was conducted once before and three times after intravenous injection. Acquisitions were obtained at 20, 90, and 180 s after injection of gadopentetate dimeglumine at a rate of 3 ml/s and a dose of 0.1 mmol/kg during the hepatic arterial, portal, and delayed phases, respectively. All detailed MRI sequences are specifically shown in **Table 1**.

**Table 1:** MRI sequences and parameters.

Parameters	Repetition time (ms)	Echo time (ms)	Section thickness (mm)	*N* excitations	Matrix	Bandwidth (Hz/pixel)	Flip angle (°)
T1-weighted imaging	6.87	2.38/4.76	4	1	320 * 240	430/490	10
T2-weighted imaging	2,400	94	5.5	2	384 * 218	194	160
Diffusion-weighted imaging	5,100	55	6	2	84 * 128	1,562	/
Contrast-enhanced imaging	4.36	2	3.5	2	320 * 195	64	10

The 1.5-T MRI imager was a Magnetom Aera (Siemens Medical Solutions) unit.

### Imaging Analysis

The MRI images were acquired, evaluated, and processed *via* a picture archiving communication system (PACS) workstation. All MRI images were reviewed by two pancreatic radiologists (with 6 and 18 years of experience, respectively), who were blinded to the pathological and clinical data. Furthermore, all images were evaluated independently by two radiologists. In the event that there is inconsistency, another more experienced observer was invited for an opinion, and a majority decision was finally reached.

Qualitative data included the following: a) tumor location (head/neck, body, or tail), b) tumor consistency (solid, cystic, or solid and cystic), c) tumor margins (well-defined/ill-defined), d) main pancreatic duct (MPD) dilation (presence/absence), e) pancreatic atrophy (presence/absence), f) bile duct (BD) dilation (presence/absence), g) infiltration of peripancreatic fat (presence/absence), h) invasion of peripancreatic vessels (presence/absence), i) lymph node invasion (presence/absence), and j) signal on T2-weighted portal venous and delayed phase MRI images (defined as hypointense or iso-/hyperintense in comparison with the surrounding normal pancreatic parenchyma). MPD dilation was defined as MPD with a diameter larger than 3 mm. Invasion of peripancreatic vessels was considered based on the following: a) a mass adjoined >90° of the vascular circumference, b) occlusion of major peripancreatic arteries, and c) a mass adjoined >180° of the circumference of the portal vein (PV) or superior mesenteric vein (SMV). Lymph node invasion was considered as having at least one or more peripancreatic lymph nodes that are larger than 1 cm in diameter. Tumor consistency was divided according to the following three types: a) solid exhibiting an enhancing solid part of >90% of the mass, b) cystic exhibiting an enhancing solid part of <50% of the mass, and c) solid and cystic exhibiting an enhancing solid portion of 50%–90% of the mass.

Quantitative data analysis included a) tumor size (the maximal diameter of the tumors) and b) apparent diffusion coefficient (ADC) values (*b* = 500 s/mm^2^). The ADC values were assessed *via* ROIs on the ADC images. All ROIs were manually drawn to include the largest part of the mass, avoiding the adjacent pancreas parenchyma, large vessels, and areas of hemorrhage or necrosis. All quantitative data were measured thrice by one experienced pancreatic radiologist, and the average values were finally used for further research.

### Pathological Analysis

Histopathologic analysis of all excised lesions was performed by two experienced pancreatic pathologists. The pathological grade of all PNETs was classified according to the 2017 World Health Organization classification as follows: G1 (low grade), G2 (intermediate grade), or G3 (high grade) (12).

### Construction and Validation of the MRI-Based Nomogram

In order to build a combined nomogram integrating various MRI features, we constructed a multivariable logistic regression model to identify the preoperatively discriminative radiological findings between non-hypervascular PNETs and PDACs and then integrated all significant features to construct a valuable discriminative radiological model. Furthermore, validation of the performance of the developed MRI-based nomogram was evaluated *via* the calibration curve and concordance index (C-index). The calibration curve was performed to graphically describe discriminative outcomes versus real outcomes, and the C-index was conducted to assess the discriminative performance of the developed MRI-based nomogram.

### Statistical Analysis

All quantitative parameters were represented as mean ± standard deviation (SD) or median [interquartile range (IQR) = 25–75], and categorical parameters were represented as number (percentage). Depending on the distribution of variables, continuous variables between two groups were analyzed *via* Student’s *t*-test or the Mann–Whitney *U* test, and categorical parameters between two groups were analyzed *via* the *χ* ^2^ test or Fisher’s exact test. The dependent discriminative parameters for differentiating non-hypervascular PNETs from PDACs were analyzed using receiver-operating characteristic (ROC) curve analysis, and then the corresponding sensitivity, specificity, and area under the ROC curve (AUC) were calculated. *κ* statistics was performed to assess the interobserver variability for categorical parameters. The grade of agreement was classified as follows: slight (*κ* < 0.20), fair (*κ* = 0.21–0.40), moderate (*κ* = 0.41–0.60), substantial (*κ* = 0.61–0.80), and outstanding (*κ* > 0.80).

Univariate and multivariate logistic regression analyses were used to identify the independent risk factors of the two groups. Then, a discriminative nomogram based on the significant MRI features was formulated *via* the rms package in R project. The C-index and the calibration curve were performed to assess the performance of the preliminary MRI-based nomogram. All statistical analyses were performed using SPSS software (version 22.0; IBM, Armonk, NY, USA) and R project (version 3.5.0). The tests were two-sided and *P*-value <0.05 was defined as statistically significant.

## Results

### Patient and Tumor Characteristics

A total of 131 patients, consisting of 44 with non-hypervascular PNETs and 87 with PDACs, were finally included in this retrospective study. There were no multifocal masses in both cohorts. In the non-hypervascular PNET group, the age of the patients (median age, 55.6 ± 14.6 years) ranged from 19 to 79, and in the PDAC group, the patients’ age (median age, 57.7 ± 12.6 years) ranged from 32 to 79. Furthermore, in this cohort, the ratio of female patients in the non-hypervascular PNET group was 65.9% (29/44), which was slightly larger than that in the PDAC group (52.3%, 46/87). Based on the 2017 WHO classification, among the non-hypervascular PNET cohort, 15 masses were classified as G1 (34.1%) and 65.9% masses were defined as G2/G3.

### Interobserver Agreement for Qualitative MRI Features

An outstanding interobserver agreement was achieved for tumor consistency (*κ* = 0.812), tumor margin (*κ* = 0.878), MPD dilation (*κ* = 0.924), pancreatic atrophy (*κ* = 0.873), BD dilation (*κ* = 0.819), and signal in T2-weighted images, portal phase, and delayed phase (*κ* = 0.846, 0.896, and 0.876, respectively), and a substantial agreement was obtained for invasion of peripancreatic vessels (*κ* = 0.798) and infiltration of peripancreatic fat (*κ* = 0.786).

### Analysis of the Predictive Factors for MR Imaging Features in Differentiating Non-Hypervascular PNETs From PDACs

**Figure 2:**
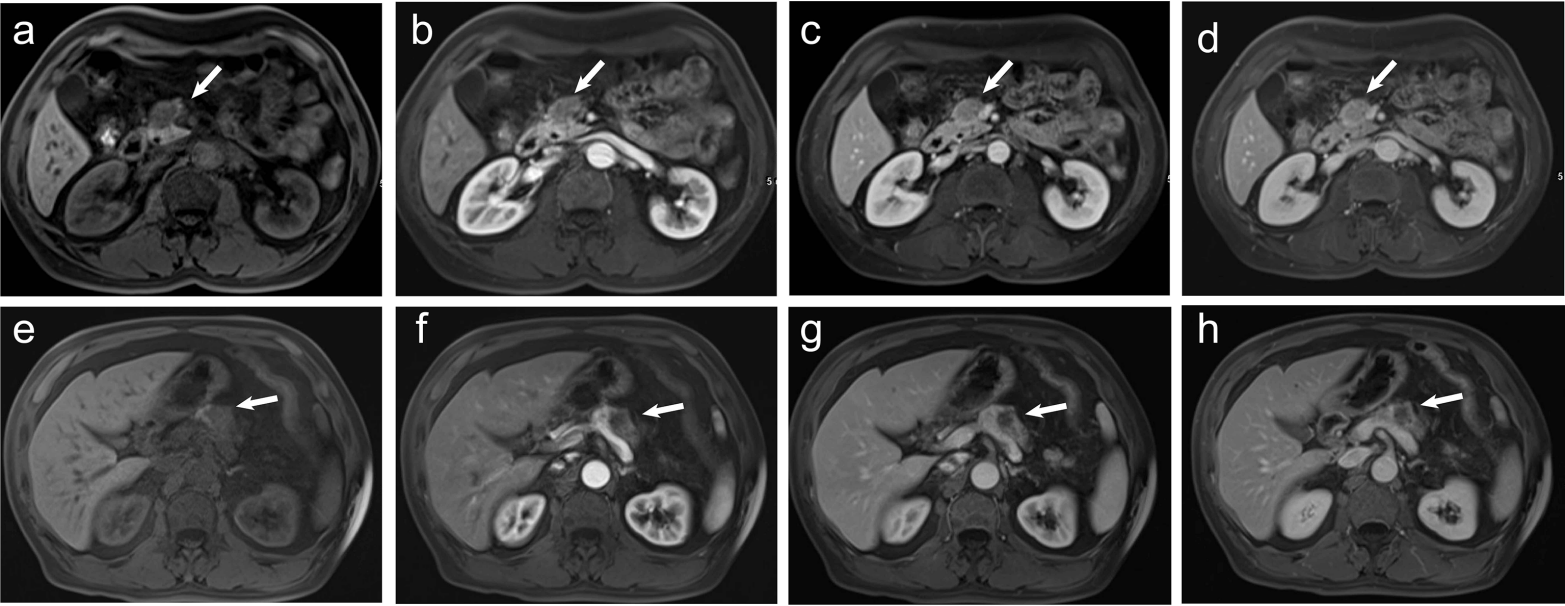
**(A–D)** A 60-year-old man pathologically diagnosed with G2 non-hypervascular PNETs. **(A)** T1-weighted imaging shows a well-defined hypointense mass in the head of the pancreas (white arrow). **(B)** The mass in the arterial phase shows hypointensity (white arrow). **(C, D)** The mass in both portal **(C)** and delayed phases **(D)** shows relative isointensity (white arrow). **(E–H)** A 60-year-old man pathologically diagnosed with PDACs. **(E)** T1-weighted imaging shows an ill-defined hypointense mass in the body of the pancreas (white arrow). **(F)** The mass in the arterial phase shows hypointensity (white arrow). **(G)** The mass in the portal phase shows obvious hypointensity with mild pancreatic duct dilation (white arrow). **(H)** The mass in the delayed phase shows obvious hypointensity (white arrow).

**Figure 3:**
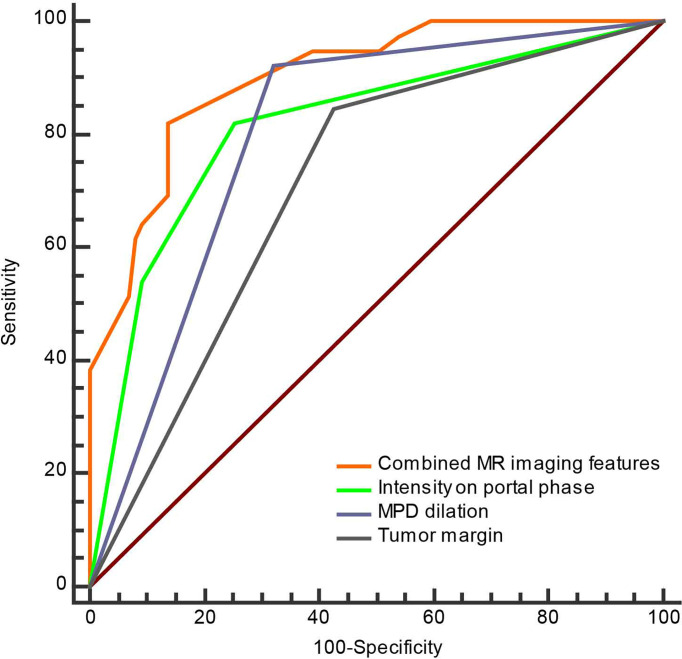
The ROC curve of separate three MRI features and combined MRI features for discrimination of the non-hypervascular PNETs from PDACs.

To investigate the predictive value of MR imaging features in differentiating non-hypervascular PNETs from PDACs, univariate and multivariate analyses were performed, and these are shown in **Tables 2**, **3**. The univariate analysis data exhibited that tumor margin (*P* < 0.001), MPD dilation (*P* < 0.001), pancreatic atrophy (*P* = 0.03), infiltration of peripancreatic fat (*P* = 0.001), invasion of peripancreatic vessels (*P* = 0.004), and signal in the portal phase (*P* < 0.001) were considered as significantly different MR imaging features between the non-hypervascular PNETs and PDACs. Next, the above significant MRI parameters were further subjected to multivariate analysis. The multivariate analysis data showed that the well-defined tumor margin (*P* = 0.012; OR: 6.622; 95% CI: 1.510, 29.028), the absence of MPD dilation (*P* = 0.047; OR: 4.309; 95% CI: 1.019, 18.227), and hyperintensity in the portal phase (*P* < 0.001; OR: 53.486; 95% CI: 10.690, 267.618) were independent risk factors for discriminating non-hypervascular PNETs from PDACs (**Figure 2**). The ROC analysis exhibited that the sensitivity, specificity, and AUC of the tumor margin, MPD dilation, intensity in the portal phase, and the combined MR imaging features were 84.62%, 57.47%, and 0.710; 92.31%, 67.82%, and 0.801; 82.05%, 74.71%, and 0.814; and 82.05%, 86.21%, and 0.900, respectively (**Figure 3**).

**Table 2:** Clinical and radiological characteristics.

	Non-hypervascular PNETs (*n* = 44)	PDACs (*n* = 87)	*P*-value
Age (years)^a^	55.6 ± 14.6	57.7 ± 12.6	0.401
Sex			0.154
Female	29 (65.91%)	46 (52.87%)	
Male	15 (34.09%)	41 (47.13%)	
Location			0.198
Head/neck	23 (52.27%)	52 (59.77%)	
Body	4 (9.09%)	2 (2.3%)	
Tail	17 (38.64%)	33 (37.93%)	
Tumor size^a^	3.3 ± 1.4	3.7 ± 1.2	0.147
Tumor consistency			0.369
Cystic	4 (9.09%)	3 (3.45%)	
Solid	34 (77.27%)	69 (79.31%)	
Solid and cystic	6 (13.64%)	15 (17.24%)	
Tumor margin			<0.001
Well-defined	33 (75%)	27 (31.03%)	
Ill-defined	11 (25%)	60 (68.97%)	
MPD dilation			<0.001
Absence	36 (81.82%)	31 (35.63%)	
Presence	8 (18.18%)	56 (64.37%)	
Pancreatic atrophy			0.030
Absence	38 (86.36%)	60 (68.97%)	
Presence	6 (13.64%)	27 (31.03%)	
BD dilation			0.082
Absence	37 (84.09%)	61 (70.11%)	
Presence	7 (15.91%)	26 (29.89%)	
Infiltration of peripancreatic fat			0.001
Absence	33 (75%)	37 (42.53%)	
Presence	11 (25%)	50 (57.47%)	
Lymph node invasion			0.351
Absence	29 (65.91%)	50 (57.47%)	
Presence	15 (34.09%)	37 (42.53%)	
Invasion of peripancreatic vessels			0.004
Absence	35 (79.55%)	47 (54.02%)	
Presence	9 (20.45%)	40 (45.98%)	
Signal in T2-weighted images			0.292
Hypointense	1 (2.27%)	1 (1.15%)	
Isointense	13 (14.94%)	16 (18.39%)	
Hyperintense	30 (68.18%)	70 (80.46%)	
Signal in the portal phase			<0.001
Hypointense	8 (18.18%)	69 (79.31%)	
Isointense	15 (34.09%)	10 (11.49%)	
Hyperintense	21 (47.73%)	8 (9.2%)	
Signal in the delayed phase			0.061
Hypointense	66 (150%)	26 (29.89%)	
Isointense	20 (45.45%)	15 (17.24%)	
Hyperintense	1 (2.27%)	3 (3.45%)	
ADC (×10^−3^ mm^2^/s)^b^	1.25 (0.81–1.49)	1.16 (0.80–1.48)	0.153

Unless otherwise indicated, data are the number of lesions, with percentage in parentheses.

a Data are expressed as mean ± standard deviation.

b Data are expressed as [interquartile range (IQR) = 25–75].

ADC, apparent diffusion coefficient; MPD, main pancreatic duct; BD, bile duct.

**Table 3:** Univariate and multivariate analyses for relevant MRI features for differentiating non-hypervascular PNETs and PDACs.

Risk factors	Univariate analysis			Multivariate analysis		
	OR	95% CI	*P*-value	OR	95% CI	*P*-value
Age (years)	0.988	0.962, 1.016	0.398			
Sex						
Female	1.723	0.812, 3.656	0.156			
Male^a^						
Location						
Head/neck^a^						
Body	4.522	0.773, 26.465	0.094			
Tail	1.165	0.543, 2.500	0.696			
Tumor size	0.776	0.572, 1.051	0.102			
Tumor consistency						
Cystic^a^						
Solid	1.232	0.439, 3.457	0.692			
Solid and cystic	3.333	0.567, 19.593	0.183			
Tumor margin (well-defined)	6.667	2.937, 15.132	**<0.001**	6.622	1.510, 29.028	**0.012**
MPD dilation (absence)	9.482	3.900, 23.053	**<0.001**	4.309	1.019, 18.227	**0.047**
BD dilation (absence)	2.253	0.890, 5.705	0.087			
Pancreatic atrophy (absence)	2.850	1.007, 7.544	**0.035**	1.947	0.423, 8.961	0.392
Infiltration of peripancreatic fat (absence)	4.054	1.814, 9.058	**0.001**	2.892	0.586, 29.028	0.192
Lymph node invasion (absence)	1.431	0.673, 3.042	0.352			
Invasion of peripancreatic vessels (absence)	3.310	1.421, 7.706	**0.006**	1.025	0.179, 5.875	0.978
Signal in T2-weighted images			0.292			
Hypointense^a^						
Isointense	1.896	0.812, 4.425	0.139			
Hyperintense	2.333	0.141, 38.548	0.554			
Signal in the portal phase						
Hypointense^a^						
Isointense	12.937	4.374, 38.268	**<0.001**	28.298	5.956, 134.463	**<0.001**
Hyperintense	22.641	7.547, 67.675	**<0.001**	53.486	10.690, 267.618	**<0.001**
Signal in the delayed phase						
Hypointense^a^						
Isointense	1.904	0.848, 4.274	0.119			
Hyperintense	7.615	0.757, 76.584	0.085			
ADC (×10^−3^ mm^2^/s)	1.993	0.771, 5.155	0.155			

a Data were utilized as the reference variable.

OR, odds ratio; CI, confidence interval; ADC, apparent diffusion coefficient; MPD, main pancreatic duct; BD, bile duct. The values provided in bold type mean ＜0.05 and statistically significant.

### Construction and Validation of the MRI-Based Nomogram for Discrimination Between Non-Hypervascular PNETs and PDACs

**Figure 4:**
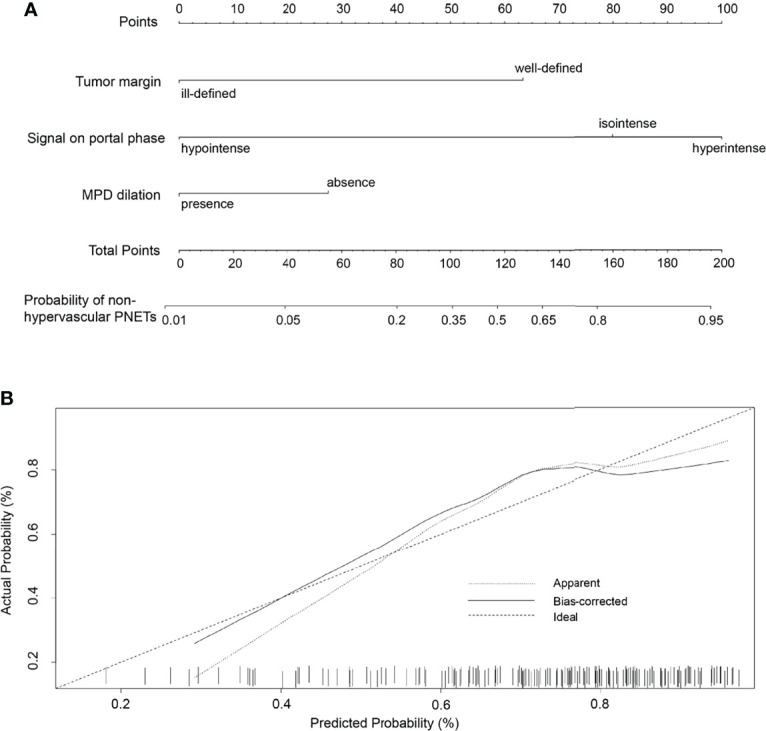
**(A)** The developed nomogram integrating three statistically significant MRI features to discriminate non-hypervascular PNETs from PDACs. **(B)** The calibration curve of the MRI-based nomogram to discriminate non-hypervascular PNETs from PDACs.

To develop a visual and individualized differential model, we have combined various significant MR imaging features in multivariate logistic regression to construct a novel nomogram for discriminating non-hypervascular PNETs from PDACs (**Figure 4A**). In this cohort, the C-index was calculated to assess the discriminative performance of various MRI features. The results represented that the C-index for differential diagnosis with the tri-combined nomogram was 0.914 (95% CI: 0.036, 0.134), which was larger than other C-indices for the other single or bi-combined variables (**Table 4**).

**Table 4:** Discriminatory capabilities of the nomogram and independent MRI features.

Factors	C-index	95% CI
Tumor margin	0.719	0.199, 0.361
Signal in the portal phase	0.817	0.109, 0.256
MPD dilation	0.731	0.193, 0.345
Nomogram incorporating (tumor margin + signal in the portal phase)	0.896	0.044, 0.162
Nomogram incorporating (tumor margin + MPD dilation)	0.779	0.147, 0.294
Nomogram incorporating (signal in the portal phase + MPD dilation)	0.874	0.066, 0.185
Nomogram incorporating (tumor margin + signal in the portal phase + MPD dilation)	0.914	0.036, 0.134

**Figure 5:**
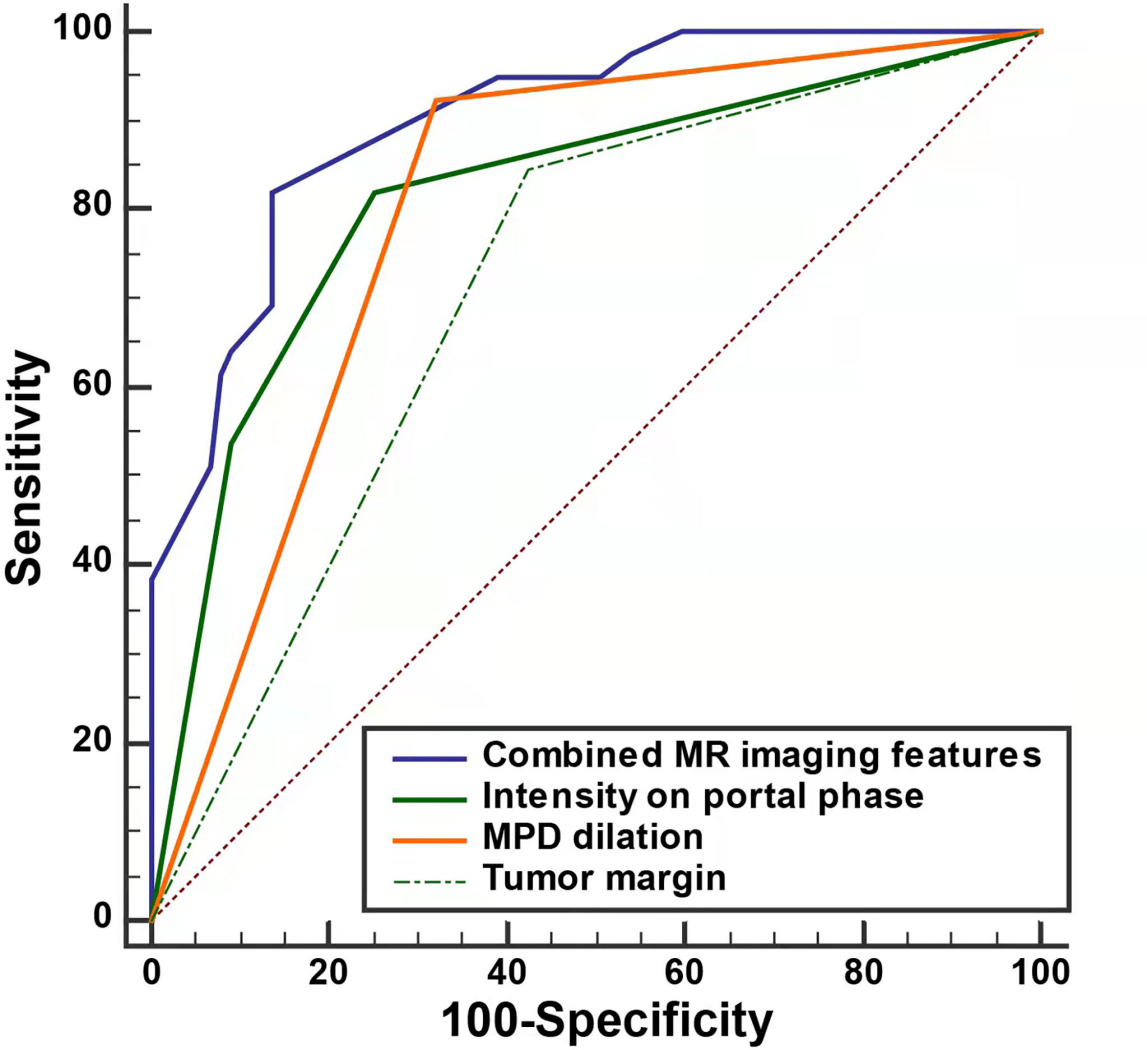
The decision curve analysis for the MRI-based nomogram. The red line displays the integrated MRI features, the blue line the signal in the portal phase, the purple line the MPD dilation, the green line the tumor margin, the gray line the hypothesis that all patients had non-hypervascular PNETs, and the black line the hypothesis that all patients had PDACs. The *x*-axis displays the high-risk threshold and the *y*-axis represents the net benefit.

To validate the discriminative effect of the developed nomogram, the calibration curve and decision curve analysis (DCA) were performed. The calibration curve results implied better consistency between estimation and observation for the discrimination performance of the two neoplasms (**Figure 4B**). Furthermore, as shown in **Figure 5**, the DCA exhibited that the developed nomogram represented better discriminative net benefits with a broader scope of threshold probabilities compared with the single MR imaging features, implying that the MRI feature-based nomogram can serve as a more effective method for differentiating non-hypervascular PNETs from PDACs.

## Discussion

In the present study, we not only investigated the performance of MRI features for discrimination of non-hypervascular PNETs and PDACs but also constructed and validated a more practical differential diagnosis model merging diverse MRI findings, which showed better diagnostic efficiency than separated MRI features. In this cohort, tumor margin, MPD dilation, and intensity in the portal phase were significantly discriminative MRI features between non-hypervascular PNETs and PDACs.

Several studies have reported that about 20%~43% of PNETs exhibited iso- or hypointensity in the arterial phase of contrast-enhanced MDCT or MRI, which is similar to the imaging findings in the arterial phase of pancreatic cancer, leading to misdiagnosis (13, 14). In our study, 26.3% (44/167) of PNET patients showed non-hyperintensity in the arterial phase of dynamic enhanced MRI, which coincided with previous studies (8). Among these cases, 68.8% of non-hypervascular PNETs were classified as G2/G3, which coincided with previous studies (8).

In this study, a well-defined tumor margin has been more often shown in the non-hypervascular PNETs than in PDACs. In a recent research by Jeon et al., the authors reported that tumor margin can serve as a discriminatively morphologic characteristic between non-hypervascular PNETs and PDACs (15). Likewise, Karmazanovsky et al. have also suggested that smooth and regular margins are more commonly observed in non-hypervascular PNETs in comparison with PDACs (58% vs. 25%) (8). This may be partly explained by the fact that non-hypervascular PNETs show less infiltration into surrounding tissues than PDACs.

In addition, the absence of MPD dilation, in our cohort, is another significant MRI feature to discriminate non-hypervascular PNETs from PDACs, which may be associated with the location of origin of the two tumors. Notably, PNETs originated from progenitor islet cells of pancreatic parenchyma. However, PDACs originated from the ductal epithelium of the pancreas, which is more likely to infiltrate into the pancreatic duct and cause dilation or obstruction of the pancreatic duct. Several recent studies have shown that the absence of MPD dilation in both CT and MRI features is more likely to occur in PNETs, which is in agreement with our research results (16, 17).

Furthermore, our study also investigated that, compared with PDACs, iso- or hyperintensity in the portal phase in the dynamic enhanced MRI more commonly appeared in non-hypervascular PNETs. A recent study has also reported that there is a significant difference in the enhancement patterns of MRI between non-hypervascular PNETs and PDACs (15). Moreover, in that study, the enhancement degree of the portal phase in the non-hypervascular PNETs was obviously higher than that in the PDACs, which is consistent with our study’s results. Similarly, in another study, hyperenhancement in the portal venous phase and persistent iso-enhancement were the significant independent CT features of non-hypervascular PNETs (8). This can be attributed to the pathological nature of PDACs (having lower vascularity and a higher rate of fibrosis compared with PNETs). Furthermore, in our cohort, other MRI features, including pancreatic atrophy, BD dilation, infiltration of peripancreatic fat, and invasion of peripancreatic vessels, are not independent predictors for the discrimination of non-hypervascular PNETs and PDACs. However, in two other recent studies, peripancreatic infiltration and pancreatic parenchymal atrophy are discriminative CT features between PNETs and PDACs, which may be caused by the different sample sizes and inclusion criteria (8, 18).

Although there are overlapping imaging findings between non-hypervascular PNETs and PDACs, the treatment strategies and prognosis are totally different. Specifically, compared with PNETs, thorough surgical approaches, such as the Whipple procedure or pylorus-preserving pancreatoduodenectomy, are more beneficial to patients. Undoubtedly, preoperative imaging discrimination of non-hypervascular PNETs from PDACs is of great importance in developing treatment strategies, improving patient outcomes (4, 15, 19). For example, previous studies reported that endoscopic ultrasound (EUS) features are correlated with malignancy of non-hypovascular solid pancreatic tumors, and EUS tissue acquisition (EUS-TA) is helpful for obtaining pathological results before surgery. However, such imaging strategies are still invasive and complex (20, 21). Thus, in this study, a preliminary MRI-based nomogram merging the various significant MRI parameters derived from multivariate regression analysis was developed for the individualized discrimination of non-hypervascular PNETs from PDACs. The developed nomogram is of great clinical significance because it eliminates the complicated equation and the calculation of the regression analysis model and enables clinicians to intuitively and graphically calculate the probability of disease (22). As far as we know, this is the first time that a radiologically user-friendly model was constructed combining diverse MR imaging findings to improve differential diagnostic performance. Furthermore, the preliminarily developed model was further assessed by the calibration curve, C-index, and DCA, to determine its practicality and accuracy. As shown by the results, the calibration curve represented a favorable coherence between the nomogram-estimated and the actually observed probability, and the C-index validated that this developed nomogram optimized the accuracy of discrimination. Meanwhile, DCA showed the best clinical benefit with a wider range of threshold probability, guaranteeing the dependability of the developed nomogram.

This study also has several limitations. First, there was inevitably inherent selection bias because only cases with surgically resected PNETs and PDACs were enrolled in this retrospective study. Second, due to some technical difficulties, this study only enrolled a relatively large sample of non-hypervascular PNETs from a single center; thus, our results may not represent the true spectrum of PNETs. Third, owing to the limitation of the number of cases, internal and external validation were not performed. Fourth, since non-hypervascular PNETs are relatively rare, we did not create a test group and a validation group nor included an external validation group. Although a very clinically valuable discrimination model was constructed in this study, it still needs to be verified by further experimental studies. Thus, to validate the results of this study in the future, a much larger sample and multicenter data should be utilized. Finally, although the practicability and accuracy of the developed model were assessed, this tool did not integrate another clinical variable. Therefore, this tool should be further improved and validated in another cohort.

In conclusion, we thoroughly investigated the significantly useful MRI features, including tumor margin, MPD dilation, and intensity in the portal phase, to discriminate non-hypervascular PNETs from PDACs. Notably, a radiologically discriminative nomogram incorporating diverse MRI parameters was constructed and validated, which may improve the efficiency and accuracy of diagnosis and provide more efficient communication among radiologists, clinicians, and patients.

## Data Availability Statement

The raw data supporting the conclusions of this article will be made available by the authors, without undue reservation.

## Ethics Statement

The studies involving human participants were reviewed and approved by the Institutional Review Board of the Affiliated Hospital of Guizhou Medical University. Written informed consent for participation was not required for this study in accordance with national legislation and institutional requirements. Written informed consent was obtained from the individual(s) for the publication of any potentially identifiable images or data included in this article.

## Author Contributions

JX and XD participated in the design of the study. JY, YF, JZ, YZ, and SC were responsible for collecting the patients’ data. JX and JY performed the statistical analysis. JX and JY wrote the manuscript. JJ and XD revised the manuscript. All authors contributed to the article and agreed to the submitted version.

## Conflict of Interest

The authors declare that the research was conducted in the absence of any commercial or financial relationships that could be construed as a potential conflict of interest.

## Publisher’s Note

All claims expressed in this article are solely those of the authors and do not necessarily represent those of their affiliated organizations, or those of the publisher, the editors and the reviewers. Any product that may be evaluated in this article, or claim that may be made by its manufacturer, is not guaranteed or endorsed by the publisher.
